# PDGF-driven proliferation, migration, and IL8 chemokine secretion in human corneal fibroblasts involve JAK2-STAT3 signaling pathway

**Published:** 2008-05-30

**Authors:** Neeraj Vij, Ajay Sharma, Mahesh Thakkar, Sunilima Sinha, Rajiv R. Mohan

**Affiliations:** 1 Mason Eye Institute, School of Medicine, University of Missouri-Columbia, Columbia, MO; 2 College of Veterinary Medicine, University of Missouri-Columbia, Columbia, MO; 3 Harry S. Truman Memorial Veterans Hospital, Columbia, MO; 4 Department of Neurology, University of Missouri, Columbia, MO

## Abstract

**Purpose:**

Platelet-derived growth factor (PDGF) is associated with corneal fibroblast migration and proliferation and plays an important role in corneal wound healing. However, the intracellular mechanisms of PDGF-mediated functions in corneal fibroblasts are poorly understood. We tested the hypothesis that PDGF functional activities in the cornea involve the Janus kinase-2/signal transducers and activators of transcription-3 (JAK2-STAT3) signaling pathway and whether PDGF induces the expression of suppressors of cytokine signaling 3 (SOCS3), belonging to the novel family of feedback regulators of cytokine and growth factor activities.

**Methods:**

Human corneal fibroblast (HSF) cultures were used as an in vitro model for functional analysis. Real-time polymerase chain reactions were performed to quantify gene expression. Immunoprecipitation and immunoblotting techniques were used to measure protein expression. Cell growth, migration, and ELISA assays were used for functional validation.

**Results:**

Low endogenous levels of STAT3 and SOCS3 mRNA and protein expression were noted in HSFs. PDGF treatment of HSF significantly induced SOCS3 mRNA (3.0–4.5 fold) and protein (1.5–2.5 fold) expression in a time-dependent manner. Similarly, PDGF treatment of HSF significantly increased STAT3 protein expression at two tested time points (2.5–2.96 fold). Cultures exposed to vehicle (control) did not show any change in SOCS3 and STAT3 mRNA or protein expression. An addition of AG-490, a selective inhibitor of the JAK2-STAT3 pathway, significantly inhibited PDGF-mediated STAT3 induction and cell growth and migration in HSF. We also observed that PDGF induced interleukin-8 (IL8) chemokine secretion (2 fold) and AG-490 inhibited IL8 secretion.

**Conclusions:**

Our data showed that PDGF induced STAT3, SOCS3, and IL8 chemokine secretion in human corneal fibroblasts. Further, PDGF-induced cell growth, migration, and IL8 secretion in corneal fibroblast involve the JAK2-STAT3 signaling pathway.

## Introduction

Corneal wound healing plays a critical role in the maintenance of corneal transparency and visual acuity. Corneal epithelial cell migration and proliferation, keratocyte apoptosis, extracellular matrix remodeling, and transdifferentiation of keratocyte to fibroblasts and myofibroblasts are involved in corneal wound healing [1-3]. These processes are largely regulated by the cytokines and growth factors through the activation of several intracellular signaling pathways including those involving mitogen-activated proteins (MAP) kinases, Janus kinases (JAKs), and signal transducers and activators of transcription (STATs) [4-6]. Recently, a family of inhibitory molecules called the suppressors of cytokine signaling (SOCS) proteins have been described [7,8]. These proteins have highly divergent amino termini, a central Src-homology 2 (SH2) domain, and a conserved COOH-terminal region known as the SOCS box. Cytokines rapidly induce SOCS expression, and SOCS proteins in turn regulate cytokine signal transduction and STAT activation negatively either through inhibition of JAK kinases or by direct binding to signaling chains in the receptor complex [7]. Mutational analysis of SOCS1 has established that both the NH_2_-terminus and SH2 domain are essential for blocking JAK activity and cytokine signaling, but the COOH-terminal SOCS-box region is not required. Furthermore, the SH2 domain is necessary but not sufficient to inhibit JAK activity [9,10]. Another SOCS protein, SOCS3, can be induced by many cytokines and growth factors such as interleukin (IL)-2, IL3, leptin, and insulin [8,9,11]. The expression of SOCS3 is rapidly induced in T cells in response to IL2 and can strongly inhibit IL2-induced STAT5 phosphorylation [11]. Unlike other SOCS proteins, SOCS3 is rapidly tyrosine-phosphorylated after IL2 stimulation, although the importance of this phosphorylation is still unclear [11]. Although SOCS proteins inhibit the JAK/STAT pathway, their function in the modulation of other signaling pathways is unknown. Recently, SOCS gene expression has been shown to be induced by many cytokines and growth factors including the platelet-derived growth factor (PDGF) [12]. Gene microarray analysis of PDGF-stimulated human corneal fibroblasts showed a significant increase in SOCS3 expression [13].

Cytokines and chemokines also regulate immune response by regulating proliferation and migration of inflammatory and other cells to help in the process of wound healing [14-17]. A proinflammatory cytokine, IL8, is produced by epithelial and fibroblast cells and plays an important role in inflammation and wound healing [18,19]. IL8 has a capacity to recruit T-cells as well as nonspecific inflammatory cells into sites of inflammation by activating neutrophils [20]. IL8 has been shown to stimulate α-smooth muscle actin production in human fibroblasts when applied to the excision wounds in chickens and causes the wounds to contract and close more rapidly [21]. Furthermore, IL8 is chemotactic for fibroblasts and accelerates their migration and can stimulate deposition of tenascin, fibronectin, and collagen I during wound healing in vivo [21]. Both human corneal keratocytes and epithelial cells have been shown to synthesize and release IL8 following cytokine stimulation and/or infection [22,23].

Platelet-derived growth factor (PDGF) is a known modulator of fibroblast cell mitosis and chemotaxis [24-26]. Earlier, we have demonstrated the expression of PDGF-AA, PDGF-BB, and PDGF-AB and their receptors in the human cornea and their role in proliferation and chemotaxis using cultured human corneal fibroblasts [27]. Out of the three tested isoforms, PDGF-BB demonstrated a significantly higher chemotactic effect compared to PDGF-AA or PDGF-AB in human corneal fibroblasts [27]. Interestingly, PDGF-AA showed strongest chemotactic response in rabbit corneal epithelial cells [28]. Because of the chemotactic role of PDGF in corneal wound healing, we hypothesized that PDGF regulates the neutrophil chemoattractant, IL8. IL8 is known to enhance corneal wound healing by chemoattracting leukocytes and fibroblasts into the wound site rapidly [29,30]. We anticipated PDGF-mediated SOCS3 overexpression as a mechanism to regulate corneal inflammation and hypothesized that PDGF regulates IL8 chemokine secretion to promote cell migration and aids in other processes of wound repair and healing. The intracellular signaling mechanisms of PDGF-induced fibroblast migration, proliferation, and corneal wound healing are poorly understood. Jester et al. [31] reported that PDGF has a synergistic effect on TGFβ-induced transformation of rabbit corneal keratocytes to myofibroblasts and proposed that PDGF is an important mediator of wound healing. A recent report on skin fibroblasts suggests that STAT3 is involved in PDGF-driven cell migration, and this process is negatively regulated by SOCS3 [32]. A significant increase in SOCS3 transcript levels by PDGF in human corneal fibroblasts noted on gene array analysis together with our previous investigations with PDGF prompted us to hypothesize that PDGF-mediated cellular proliferation and migration in cornea involve JAK/STAT pathway under negative feedback regulation by SOCS3. Using cultured human corneal fibroblasts, we showed that PDGF-induced cellular proliferation and migration involves JAK2-STAT3 pathway under the regulation of SOCS3.

## Methods

### Cell culture

Donor human corneas were procured from the Hartland Eye Banks (St. Louis, MO). Primary human corneal fibroblasts (HSF) were generated from donor human corneas using a method described previously [33]. Briefly, the cornea was washed with an HSF medium, and the epithelium and endothelium were removed by gentle scraping with a scalpel blade. The corneal stroma was cut into small pieces and incubated in humidified CO_2_ incubator at 37 °C in Dulbecco’s modified Eagle’s medium (DMEM) containing 10% fetal bovine serum to obtain HSF. Seventy percent confluent cultures of HSF (passages 1–3) were used for experiments. To investigate the effect of PDGF, a mixture of PDGF containing equal amounts of PDGF-AA, PDGF-AB, and PDGF-BB was used for all experiments. Cultures were exposed to the mixture of PDGF (AA, AB, and BB) at a final concentration of 20 ng/ml for 1 h or 8 h. The JAK-STAT inhibitor, AG-490, was used at 100 μM final concentration for the indicated time to inhibit downstream signaling. For all the experiments, cultures were serum-starved for 24 h before being exposed to the cytokine and/or the inhibitor. The cytokines were bought from R&D System (Minneapolis, MN), and the AG-490 inhibitor was purchased from Calbiochem (San Diego, CA).

### RNA extraction, cDNA synthesis, and quantitative real-time polymerase chain reaction

Total RNA from the cells was extracted using an RNeasy kit (Qiagen Inc., Valencia, CA) and was reverse transcribed to cDNA (Promega, Madison, WI) following the vendor’s instructions using the ImProm-II Reverse Transcription kit. Real-time polymerase chain reaction (PCR) was performed using the iQ5 real-time PCR Detection System (Bio-Rad Laboratories, Hercules, CA). Fifty microliters of a reaction mixture containing 2 µl cDNA (250 ng), 2 µl forward primer (5′-TGG CGA AGG AAA TGG TCA CA-3′; 200 nM), 2 µl reverse primer (5′-GGT GAC TGT CCC GGA GGA GA-3′; 200 nM), and 25 µl 2X iQ SYBR Green Supermix (Bio-Rad Laboratories) was run at universal cycle (95 °C for 3 min, 40 cycles of 95 °C for 30 s followed by 60 °C for 60 s) according to the manufacturer’s instructions. The β-actin forward primer (5′-CGG CTA CAG CTT CAC CAC CA-3′) and reverse primer (5′-CGG GCA GCT CGT AGC TCT TC-3′) were used as a housekeeping gene. The threshold cycle (Ct) was used to detect the increase in the signal associated with an exponential growth of PCR product during the log-linear phase. The relative expression was calculated using the following formula, 2^-ΔΔCt^. The ΔCt validation experiments showed similar amplification efficiency for all templates used (the difference between linear slopes for all templates is less than 0.1). Three independent experiments were performed and the average (±SEM; standard error of the mean) results are presented in graphic form.

### Immunoprecipitation and immunoblotting

Cells were washed three times in ice-cold PBS and lysed directly on plates using M-PER protein lysis buffer containing protease inhibitor cocktail (Pierce Biotech, Rockford, IL). To immunoprecipitate SOCS3, we used 1000 µg/ml of total corneal fibroblast protein extracts and incubated them with 50 µl of protein A/G agarose beads (Santa Cruz Biotechnology Inc., Santa Cruz, CA) for 3 h at 4 °C. After preclearing, 5 µl of 1 mg/ml SOCS3 goat polyclonal antibody (Santa Cruz Biotechnology Inc.) was added to each treatment group and 5 µl of pre-immune serum served as the negative control [34]. Protein A/G agarose beads (50 µl) were added to each tube after 1 h followed by overnight incubation at 4 °C. Beads were washed once with lysis buffer (20 mM Tris-HCl, pH 7.6; 150 mM NaCl; 0.5% Triton X-100; and 10 µM phenylmethylsulfonyl fluoride) followed with two washes with PBS. The samples were suspended in Laemmli’s sample buffer (30 µl), vortexed for 1 min, centrifuged for 5 min at 10,000x g, and boiled for 5 min. For immunoblotting, the protein extracts were suspended in 30 µl of Laemmli’s sample buffer containing β-mercaptoethanol, resolved by 4%–10% SDS–PAGE, and transferred to a 0.45-µm pore size nitrocellulose membrane (Invitrogen, San Diego, CA) [35]. The STAT3, SOCS3, and β-actin antibodies were purchased from Santa Cruz Biotech. The anti-mouse, rabbit- or goat-HRP, or AP antibodies were from Amersham (Piscataway, NJ) or Santa Cruz Biotech.

### Interleukin-8 ELISA

Corneal fibroblast cultures (~70% confluence) were serum-starved for 24 h and stimulated with PDGF for 8 h. At end-time points, supernatants were collected from three independent experiments and IL8 levels were measured using solid-phase amplified sensitivity immunoassay as specified by the manufacturer (ELISA system, BioSource Europe S.A, Nivelles, Belgium). Standards and cytokine controls were included. The plates were read at 450 nm on a 96-well microplate reader (Molecular Devices, Sunnyvale, CA) using SOFT-MAX-Pro software (Molecular Devices). The mean blank reading was subtracted from each sample and control reading. A standard curve was plotted, and an IL8 concentration in each sample was determined by interpolation from the standard curve [36]. The data represents the mean of three independent experiments ±SEM .

### Cell proliferation assay

To assess the effect of PDGF on corneal fibroblast proliferation, a viable cell number was counted using a 3-[4,5-dimethylthiazol-2-yl]-2,5-diphenyl tetrazolium bromide (MTT)-based method as described previously [37]. The assay utilizes a tetrazolium compound, MTT (Sigma-Aldrich, St Louis, MO), that is bio-reduced by viable cells to a purple colored formazan product (proportional to the number of viable cells), which can be detected by measuring its absorbance at 570 nm. Using a 96 well plate, 2x10^3^ cells were plated in each well in 100 μl of DMEM, and 10 μl of MTT reagent was added to each well. Wells containing media alone without cells served as the negative controls. The plates were incubated for 2 h at 37 °C in a humidified 5% CO_2_ incubator. To study the effect of PDGF on cell growth, 20 ng/ml of PDGF (an equal mixture of AA, AB, and BB) was added in each well. Four replicates of each treatment were used for each experiment. Absorbance was recorded at 570 nm using a 96 well plate reader (BioTEk FLx 800, Winooski, VT).

### Cell migration assay

The 48 well Boyden chemotaxis chamber apparatus (Neuro Probe, Gaithersburg, MD) was used for the chemotaxis assay using polyvinyl pyrrolidine-free polycarbonate filters with a thickness of 10 μm and pore size of 8 μm. Cell migration assay was performed as previously described [27]. Briefly, polycarbonate filters were precoated with collagen type I solution (Sigma-Aldrich, St. Louis, MO). Thirty microliters of DMEM having no cytokine or cells was placed in the lower chamber of the Boyden apparatus, and after placing the polycarbonate filter, 50 μl of DMEM containing 20 ng/ml of PDGF and 15,000 HSF was placed in the upper chamber. The apparatus was incubated in humidified CO_2_ at 37 °C for the desired duration. The total number of cells migrated to the lower side of the membrane were counted from three independent experiments at 1 h and 8 h time points following methods reported earlier [27].

### Image and statistical analysis

The results were expressed as mean±standard error of the mean (SEM) of three to four replicates as indicated. Statistical analysis was performed using two-way analysis of variance (ANOVA) followed by Bonferroni multiple comparisons test for real-time PCR and cellular proliferation data. The cellular migration and IL8 ELISA results were analyzed using one-way ANOVA followed by Tukey’s multiple comparison test. A p-value less than 0.05 was considered significant. The gel data was analyzed using Image J 1.38 X image analysis software (NIH, Bethesda, MD).

## Results

### Platelet-derived growth factor mediates suppressor of cystokine-3 induction in corneal fibroblasts

**Figure 1:**
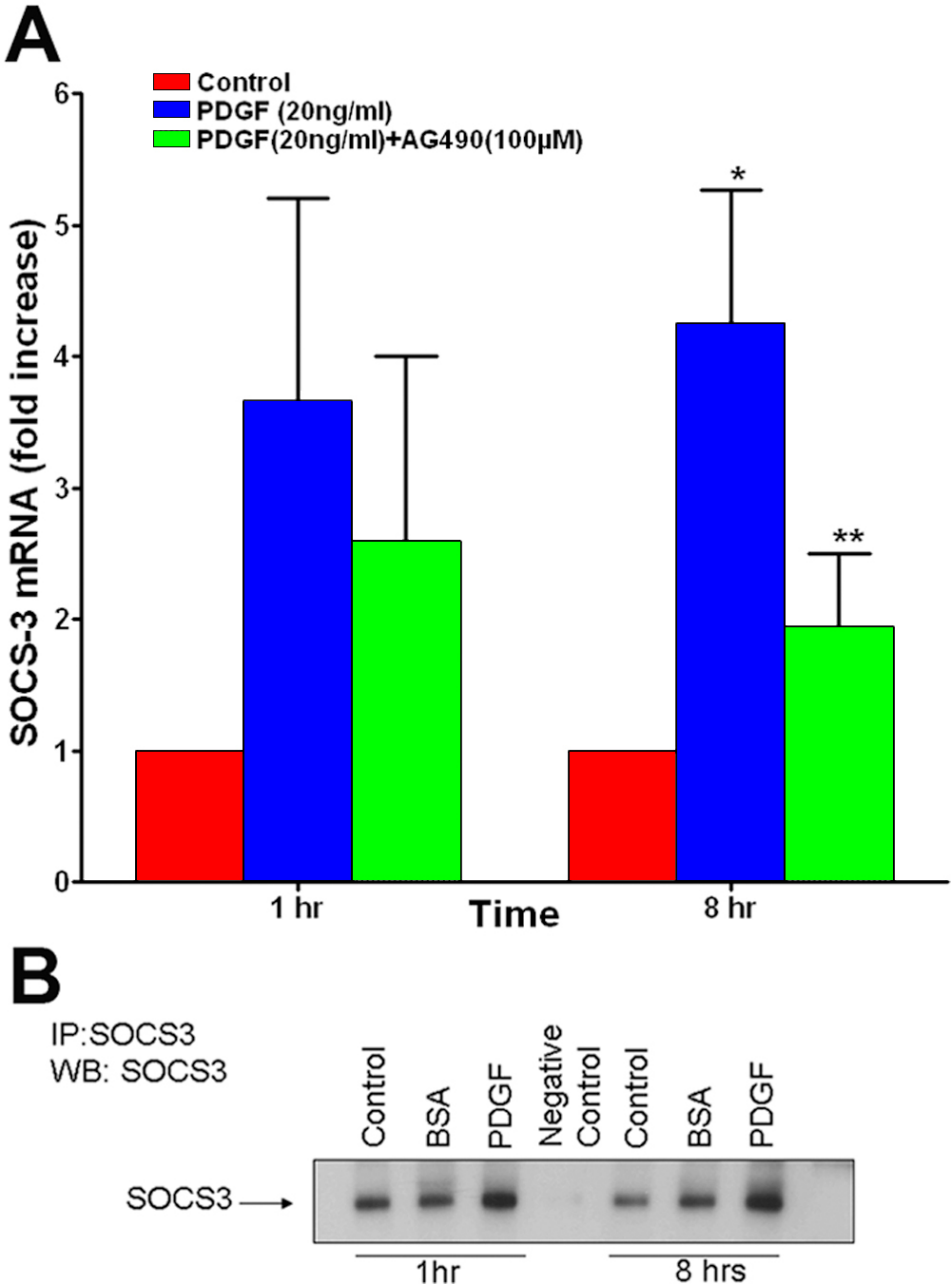
PDGF-mediated SOCS3 induction in human corneal fibroblasts involves JAK2-STAT3 pathway. Human corneal fibroblasts were exposed to a mixture of PDGF (AA, AB, and BB) at a final concentration of 20 ng/ml with or without JAK2-STAT3 inhibitor, AG-490 (final concentration of 100 μM) for 1 h or 8 h. SOCS3 transcript levels were quantified by real-time PCR, and protein levels were determined by immunoprecipitation and immunoblotting using the SOCS3 polyclonal antibody. **A**: The fold change in SOCS3 transcript levels compared to untreated control is shown as mean ±SEM. The PDGF treatment increases SOCS3 mRNA levels by threefold at 1 h and 4.5 fold at 8 h, and AG-490 treatment inhibits PDGF-induced *SOCS3* mRNA levels significantly. The asterisk indicates a p<0.05 versus control and the double asterisk indicates a p<0.05 versus PDGF. **B**: SOCS3 protein levels in PDGF-treated, BSA-treated or untreated cells showing 1.5 to 2.5 fold increase in SOCS3 protein expression by PDGF treatment. Pre-immune serum was used as a negative control for immunoprecipitation.

We quantified the suppressor of cystokine-3 (SOCS3) mRNA and protein levels in human corneal fibroblasts by real-time PCR and immunoprecipitation, respectively. The quantitative PCR detected low endogenous SOCS3 expression in HSF, and PDGF treatment resulted in a threefold increase in SOCS3 RNA expression at 1 h and 4.25 fold increase at 8 h (Figure 1A). The addition of AG-490 (100 μM), a selective inhibitor of JAK2-STAT3 pathway, caused 29% and 45% (p<0.05) reduction in PDGF-induced SOSC3 levels at 1 h and 8 h, respectively (Figure 1A). These results were in agreement with the findings of the PDGF-exposed HSF cDNA analysis carried with Affymetrix HG-U133 GeneChip system [13]. The PDGF-stimulated HSF samples revealed through immunoprecipitation a 1.5 fold and 2.5 fold increase in SOCS3 protein levels at 1 h and 8 h, respectively (Figure 1B). The vehicle-treated control samples did not show any change in SOCS3 mRNA (Figure 1A) or protein (Figure 1B) expression.

### Platelet-derived growth factor-driven STAT3 induction is JAK2-STAT3 mediated

**Figure 2:**
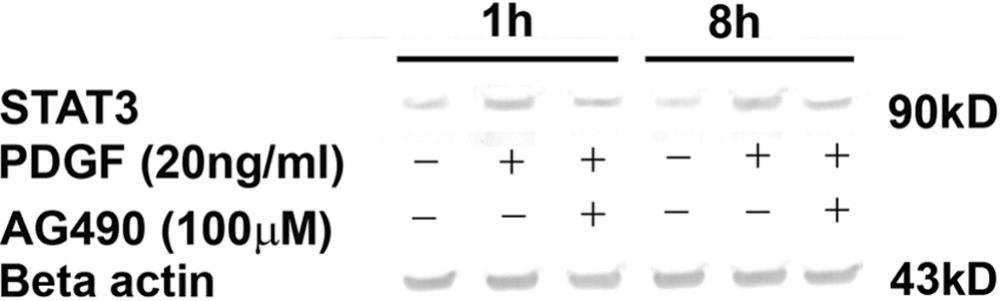
PDGF-mediated STAT3 induction in human corneal fibroblasts involves JAK2-STAT3 pathway. Human corneal fibroblasts were induced with the mixture of PDGF (AA, AB, and BB) at a final concentration of 20 ng/ml for 1 h or 8 h in the presence or absence of 100 μM AG-490. Protein levels were measured by immunoblotting using a polyclonal STAT3 antibody. PDGF treatment significantly increased STAT3 protein expression by 2.5 fold (1 h) to 2.96 fold (8 h). β-Actin immunoblot shows the equal loading in each lane.

Activation of STAT3 is known to induce SOCS3 expression as a negative feed back mechanism. To test the hypothesis that PDGF-induced SOCS3 increase is STAT3-mediated in HSF, we determined the changes in the STAT3 levels in HSF treated with PDGF. Western blot analysis of STAT3 demonstrated 2.5 fold and 2.96 fold increases in STAT3 levels at 1 h and 8 h, respectively, in the PDGF-stimulated HSF (Figure 2). The addition of AG-490 (100 μM), a selective inhibitor of JAK/STAT pathway, caused about 40% reduction in PDGF-induced STAT3 levels in HSF (1.6–2.0 fold; Figure 2).

### Platelet-derived growth factor induces interleukin-8 chemokine secretion via JAK2-STAT3 pathway

**Figure 3:**
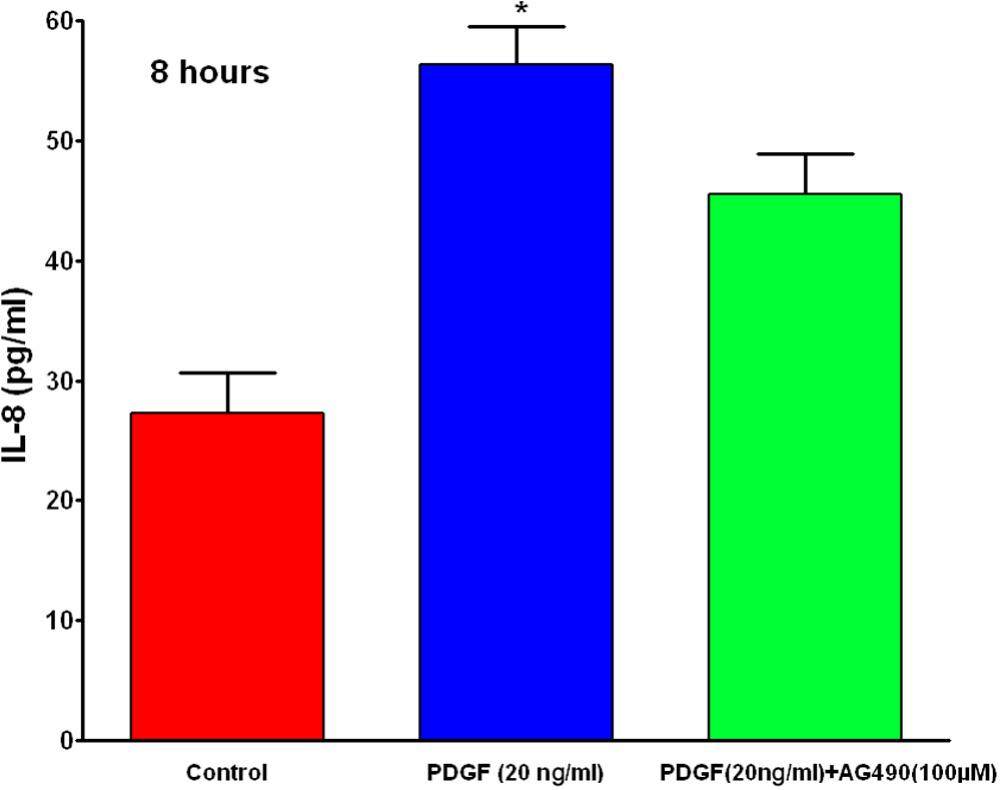
PDGF induces IL8 chemokine secretion via JAK2-STAT3 pathway. Human corneal fibroblast cultures were serum-starved for 24 h and stimulated with PDGF in the presence or absence of JAK2-STAT3 inhibitor, AG-490, for 8 h, and IL8 chemokine levels in supernatants were measured by solid-phase sandwich ELISA. PDGF induced IL8 chemokine secretion (twofold), and AG-490 significantly inhibits IL8 secretion. Data are shown as pg/ml concentration. The asterisk indicates a p<0.01 versus control.

The role of PDGF in corneal wound healing [27,28,31] and IL8-mediated neutrophil chemotaxis [12,20] has been previously documented. IL8 enhances healing by rapidly chemoattracting leukocytes and fibroblasts into the wound site [19]. We observed that PDGF increases IL8 chemokine secretion twofold in human corneal fibroblasts, indicating that IL8 is involved in PDGF-mediated corneal wound healing. We also observed that AG-490, a selective JAK/STAT inhibitor, significantly suppressed the PDGF-induced IL8 chemokine secretion levels in HSF (Figure 3). We did not detect complete blocking of PDGF-mediated IL8 secretion at 100 μM concentration of AG-490. This could be due to the lack of PDGF-mediated SOCS3 induction in the presence of AG-490 (Figure 1) or the presence of alternative pathway(s) for PDGF-mediated IL8 signaling.

### Platelet-derived growth factor mediates cell growth and migration via JAK2-STAT3 pathway

**Figure 4:**
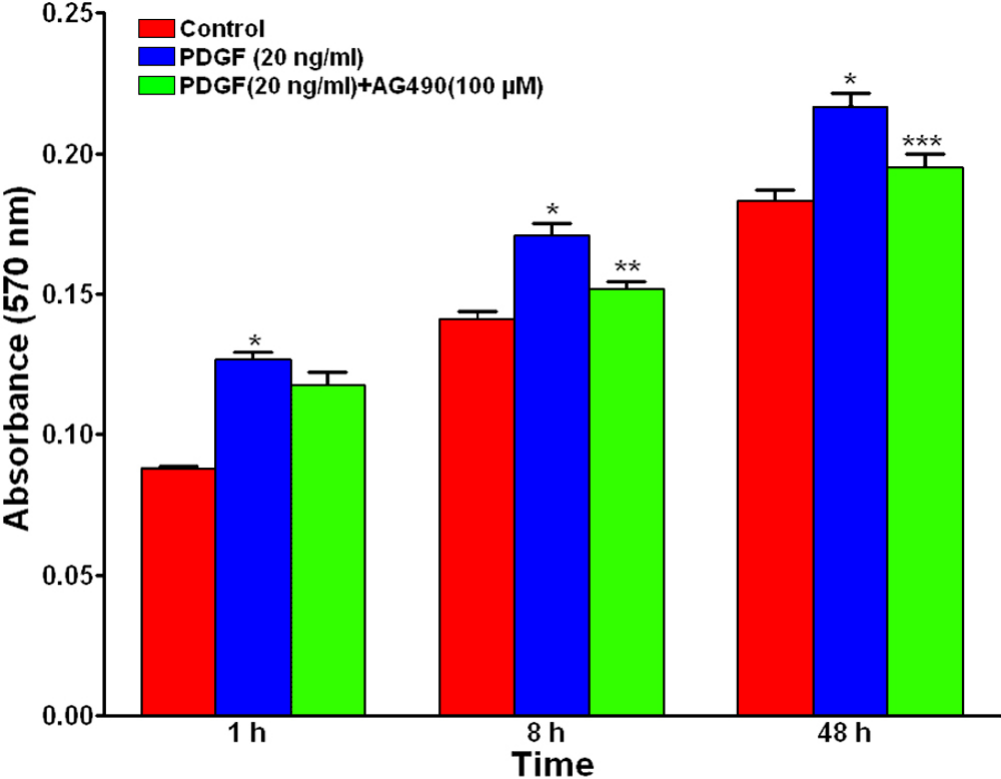
PDGF-induced cell proliferation involves JAK2-STAT3 pathway. Human corneal fibroblasts were exposed to PDGF in the presence or absence of JAK2-STAT3 inhibitor, AG-490, for 1 h, 8 h, or 48 h, and cell proliferation was quantified using the MTT assay. PDGF induced cell proliferation significantly in HSF, and AG-490 showed significant suppression of PDGF-induced cell proliferation. Data are shown as mean ±SD of absorbance at 570 nm. The asterisk indicates a p<0.001 versus control, the double asterisk indicates a p<0.01 versus PDGF, and the triple asterisk indicates a p<0.001 versus PDGF.

**Figure 5:**
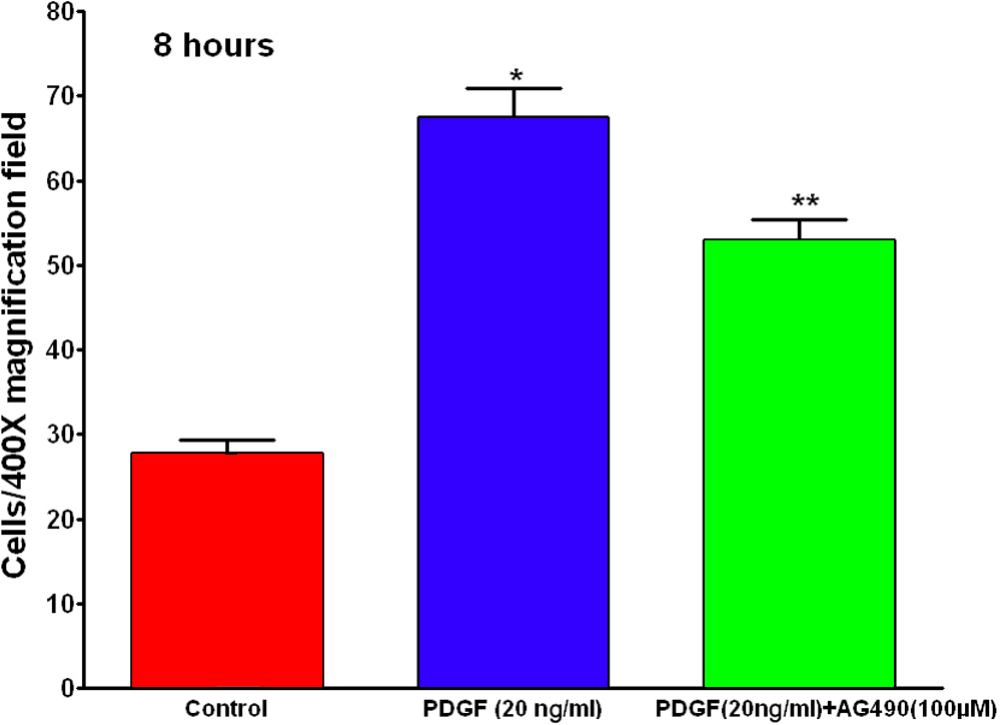
PDGF-induced cell migration involves JAK2-STAT3 pathway. Human corneal fibroblasts were exposed to PDGF in the presence or absence of JAK2-STAT3 inhibitor, AG-490, for 8 h, and cell migration was quantified using the Boyden chamber chemotaxis assay. PDGF-induced corneal fibroblast migration was inhibited significatly by AG-490. The asterisk indicates a p<0.001 versus control and the double asterisk indicates a p<0.01 versus PDGF.

Human corneal fibroblast cultures exposed to PDGF demonstrated 19%–30% increase in corneal fibroblast proliferation at 1 h (30%, p<0.001), 8 h (21%, p<0.001), and 48 h (19%, p<0.001; Figure 4). As seen in Figure 4, treatment of AG-490 (100 μM) significantly blocked PDGF-induced cell growth in HSF at 1 h (36%, not significant), 8 h (63%, p<0.01), and 48 h (68%, p<0.001). Additionally, PDGF treatment increased corneal fibroblast migration at two tested time points (1 h and 8 h). It caused a 142% (p<0.001) increase in corneal fibroblast migration at 8 h, and AG-490 (100 μM) treatment inhibited 37% (p<0.01) of the PDGF-induced cell migration at this time point (Figure 5). However, no significant change in cell migration in response to PDGF was noted at 1 h (data not shown). This data suggest that JAK2-STAT3 signaling pathway is involved in PDGF-induced corneal fibroblast proliferation and migration (Figure 4 and Figure 5).

## Discussion

PDGF and their receptors are expressed in the cornea, and its role in corneal wound healing is well established [16,27,31]. However, the underlying mechanism through which PDGF acts in the cornea is largely unknown. PDGF, upon binding to the receptors, can activate numerous signaling pathways including the tyrosine kinases from JAK and transcription factors from the family of STAT proteins [38-43]. Binding to tyrosine kinase family receptors leads to dimerization, auto phosphorylation of tyrosine residues, and recruitment of a variety of Src-homology 2 domains containing intracellular proteins, which activate downstream pathways such as mitogen activated protein (MAP) kinase, JAK/STAT, and PI3 kinase [38,41,42]. Multiple reports showed that STAT signals are under stringent regulation by a family of endogenous negative feedback regulators generically called SOCS [44-46]. SOCS proteins bind to tyrosine-phosphorylated receptors and non-receptor tyrosine kinases and prevent recruitment of STATs to the activated receptor complex [44-46]

**Figure 6:**
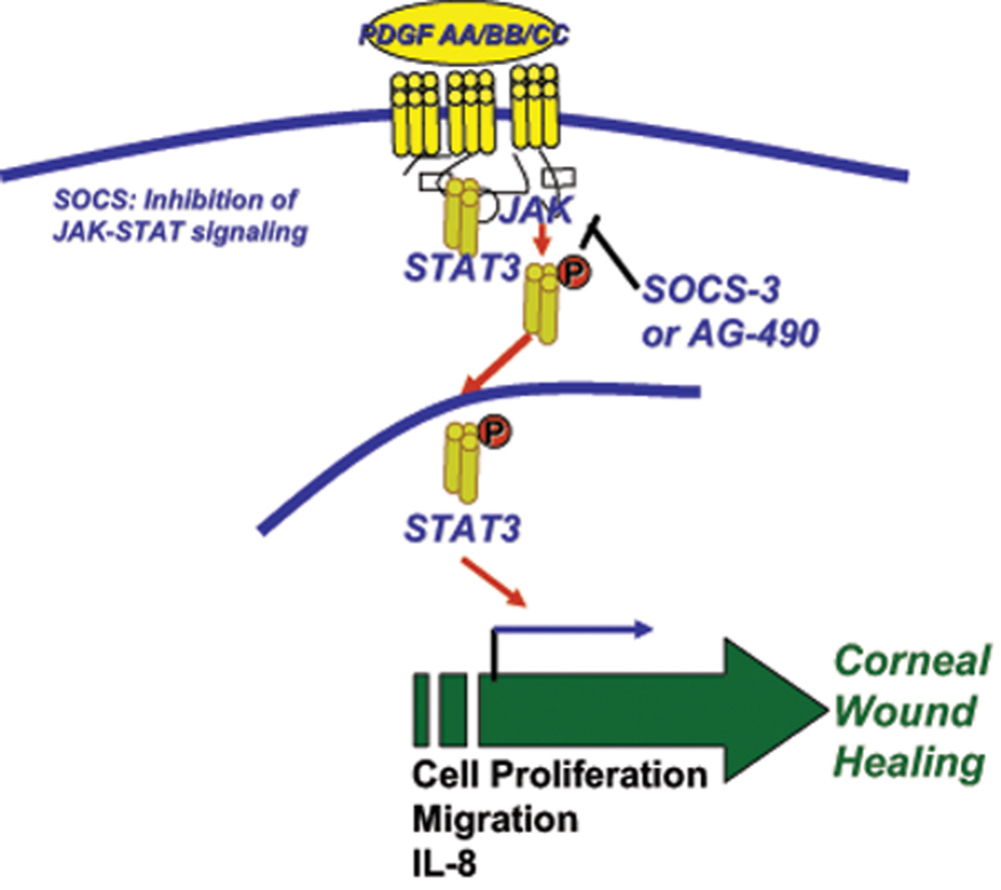
Schematic of PDGF-mediated corneal wound healing. PDGF secreted at the site of the corneal wound induces its receptors on the corneal fibroblast that induces JAK2 followed by STAT3 phosphorylation and nuclear translocation. STAT3 induces the transcription of genes involved in cell growth, migration, and IL8 chemokine secretion resulting in the initiation of corneal wound healing. SOCS3 serves as an endogenous regulator of JAK2-STAT3 inhibition as seen with the chemical inhibitor, AG-490, in vitro.

Although the role of PDGF in corneal fibroblast proliferation and migration has been previously reported [27], it is unknown whether effects of PDGF in the cornea are mediated through the activation of JAK2-STAT3 pathways and whether its activity in this ocular tissue is regulated by SOCS. Moreover, both PDGF and IL8 are chemoattractant and contribute in corneal wound healing, but the role of PDGF in IL8-mediated chemotaxis is not described [1,16,17]. This is a first report to demonstrate that PDGF induces IL8 chemokine secretion in human corneal fibroblasts. The induction of IL8 facilitates an early innate immune response to infection in the corneal stroma and represents an elementary defense mechanism in corneal wound healing [29]. IL8 enhances healing by rapidly chemoattracting leukocytes and fibroblasts into the wound site, stimulating the latter to differentiate into myofibroblasts. In turn, myofibroblasts are critical for wound contraction and closure and for the production of extracellular matrix molecules, which leads to precocious development of granulation tissue [21,47,48]. Our data suggest that PDGF-mediated IL8 chemokine secretion and cell growth and migration in corneal fibroblasts occur via JAK2-STAT3 pathway. We observed that the JAK2-STAT3 inhibitor (AG-490) inhibits PDGF functional activity in human corneal fibroblasts. A high (100 µM) concentration of AG-490 did not completely block the PDGF functional activity, suggesting the possibility of additional regulators in PDGF-induced cell proliferation, migration, and IL8 secretion. The role of signaling pathways like Ras-ERK, Akt, and PIP3-PKC in PDGF-mediated cell proliferation and migration has been shown earlier [49,50]. We observed that PDGF induces both STAT3 and SOCS3 expression and that the JAK2-STAT3 inhibitor suppresses SOCS3 expression in human corneal fibroblasts. This observation suggests that PDGF activity in the cornea may be under the negative feedback regulation of SOCS3 (Figure 6).

We have previously shown the role of STAT1 in keratocyte apoptosis that occurs in response to corneal epithelial injury using STAT1 null mice and proposed that STAT1 could be a therapeutic target for transient inhibition of keratocyte apoptosis to modulate corneal wound healing [51]. Our gene array study showed time-dependent regulation of SOCS3 in human corneal fibroblasts stimulated with PDGF. Our study results suggest that SOCS3 has a role in PDGF-mediated wound healing in the cornea [13]. Nagai et al. [32] demonstrated the role of STAT3 and SOCS3 in skin fibroblast cell migration. These authors noted nearly complete inhibition of PDGF-mediated STAT3 phosphorylation and over 50% decrease in cell migration in the skin fibroblasts that were overexpressing SOCS3. Using the transgenic animal approach, Akira and his coworkers reported that STAT3 modulates numerous biological activities including apoptosis, cell growth, and migration [52]. We report that PDGF-mediated cell growth and migration in human corneal fibroblast involves STAT3-SOCS3.

IL8 gene transcription is induced by various proinflammatory stimuli such as lipopolysaccharide, IL1β and tumor necrosis factorα [34]. The half-life of IL8 mRNA in human corneal fibroblast has been reported to be less than 1.5 h while neuropeptides like calcitonin gene-related peptide and substance P have been shown to enhance its half-life by increasing its mRNA stability [53]. Proinflammatory stimuli are considered to be a major regulator of IL8 steady-state levels in response to injury. IL8 has been shown to be involved in many of the wound healing processes. It not only serves as a chemotactic factor for leukocytes and fibroblasts but also stimulates fibroblast differentiation into myofibroblasts and promotes angiogenesis [47,48]. More studies are required to investigate other components modulated by the IL8 in corneal wound healing. Based on the involvement of JAK2-STAT3 pathway in PDGF-mediated corneal wound healing, we predict that PDGF-induced IL8 chemokine secretion could be one of the mechanisms that might be a critical mediator of wound healing in the cornea.

In summary, this study demonstrates the mechanism of PDGF-mediated corneal wound healing in vitro. The PDGF induces the proliferation, migration, and IL8 chemokine secretion in human corneal fibroblasts in part through the JAK2-STAT3 signaling pathway. Additionally, we observed that PDGF induces the expression of SOCS3 as a potential mechanism to negatively regulate inflammatory signaling during corneal wound healing.
